# Improvement of neurological recovery in the insomnia rats by Warming Yang Strategy through targeting SIRT4 by inhibiting inflammation and apoptosis

**DOI:** 10.1002/iid3.964

**Published:** 2023-08-08

**Authors:** Yuanyuan Liu, Kaihua Rao, Zhengfeng Li, Chunhua Huang

**Affiliations:** ^1^ Department of Cardiology, Jiangxi Provincial People's Hospital The First Affiliated Hospital of Nanchang Medical College Nanchang China; ^2^ Department of Neurology Affiliated Hospital of Jiangxi University of Traditional Chinese Medicine Nanchang China; ^3^ Department of Endocrinology Affiliated Hospital of Jiangxi University of Traditional Chinese Medicine Nanchang China

**Keywords:** chlorophenylalanine, insomnia, SIRT4, Traditional Chinese Medicine, Warming Yang Strategy

## Abstract

The incidence rate of insomnia is increasing, but the mechanism of it remains unclear. Warming Yang Strategy (WY) is a kind of Traditional Chinese Medicine, and it is proved to be effective in treating insomnia patients. The insomnia animal was established with chlorophenylalanine (PCPA). Morris water maze and open field test were performed to evaluate the influence of WY on the neurological recovery of insomnia rats. TUNEL staining and flow cytometry were used to measure apoptosis level. WY promoted the neurological recovery in the insomnia rats through Morris water maze and open field test evaluation. The increase of γ‐aminobutyric acid, dopamine, 5‐hydroxytryptamine, and norepinephrine caused by WY was suppressed by siSIRT4. The decrease of apoptosis and inflammation factors expression induced by WY was promoted by siRNA‐SIRT4 (siSIRT4). WY improve neurological recovery in the insomnia rats through SIRT4 by inhibiting inflammation and apoptosis. This research might provide a novel insight for the prevention and treatment of insomnia through targeting SIRT4.

## Graphical abstract

Warming Yang Strategy improve neurological recovery in insomnia rats through SIRT4 by inhibiting inflammation and apoptosis. This research might provide a novel insight for the prevention and treatment of insomnia through targeting SIRT4.

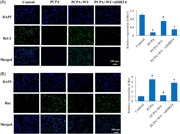

**Abbreviations**
5‐HT: 5‐hydroxytryptamine
Akt: protein kinase B
ASC: apoptosis‐associated speck‐like protein containing a caspase recruitment domain
Bcl‐2: B‐Cell CLL/Lymphoma 2
BSA: bovine serum albumin
COX2: Cyclooxygenase‐2
DAB: diaminobenzidine
DAPI: 4',6‐diamidino‐2‐phenylindole
FBS: fetal bovine serum
FITC: fluorescein isothiocyanate
GABA: γ‐aminobutyric acid
GAPDH: glyceraldehyde 3‐phosphate dehydrogenase
HRP: Horseradish peroxidase
ICAM‐1: intercellular adhesion molecule‐1
mmp9: matrix metalloproteinase‐9
NF‐κB: nuclear factor kappa B
NLRP3: nod‐like receptor family, pyrin domain containing 3
Nrf2: nuclear factor erythroid 2‐related factor 2
PBS: phosphate‐buffered saline
PCPA: chlorophenylalanine
PI3K: phosphatidylinositol 3 kinase
PVDF: polyvinylidene fluoride
RIPA: radio immunoprecipitation assay
ROS: reactive oxygen species
RPMI: roswell park memorial institute
SDS‐PAGE: sodium dodecyl sulfate‒polyacrylamide gel electrophoresis
siSIRT4: siRNA‐SIRT4
SPSS: statistical package for the social sciences
TdT: terminal deoxynucleotidyl transferase
WY: Warming Yang Strategy

## 1 INTRODUCTION

Insomnia is one of the common sleep disorders in adults. The global rate of sleep disorders is 27%, and the incidence of insomnia in Chinese adults is even more than 30%.^1^ The pathological causes of insomnia are complex. At the central level, neuroinflammation and neuronal apoptosis are believed to be novel mechanisms.^2^, ^3^ People with shorter sleep time (less than 5 or 6 h of sleep per night) or longer sleep time (more than 9 or 10 h of sleep per night) are associated with higher levels of inflammation factors compared with people with regular sleep time.^4^ It was reported that short sleep time was related to peripheral inflammasome disorder in patients with chronic insomnia. Total sleep time is negatively correlated with the levels of nod‐like receptor family, pyrin domain containing 3 (NLRP3), Apoptosis‐associated speck‐like protein containing a caspase recruitment domain (ASC), interleukin (IL)‐18, and IL‐1β.^5^

Traditional Chinese Medicine has been proved to show potential effectiveness for the prevention and treatment of nervous system diseases including neurological function injury induced by insomnia. Warming Yang Strategy (WY) comprises of six medicinal herbs including *cooked aconite*, *dried ginger*, *roasted licorice*, *cinnamon twig*, *keel*, and *oyster*. The application of WY in insomnia patients have been proved to be effective.^6^ In addition, research related with other function of WY had been reported. It was reported that WY had significant effect on improving left ventricular in rats with heart failure,^7^, ^8^ promoted the bone marrow stromal cells to proliferate and to differentiate into osteogenic, cartilage, and nerve cells.^9^ The potential function mechanism in which WY improve insomnia remains unclear.

The inflammatory reaction of nervous system is the direct inducement of neuronal apoptosis. Continuous neuroinflammatory reaction can induce neuronal apoptosis and cause many central injuries.^10^ Some neurobiological studies have shown that there is a correlation between neuronal apoptosis and insomnia.^11^ Some anti‐insomnia drugs present neuroprotective and antiapoptotic effects on neurons.^12^

The regulatory roles of SIRT4 in inflammation response and apoptosis have been reported. SIRT4 could upregulate proteoglycan and collagen II, but inhibit reactive oxygen species (ROS) and inflammatory response.^13^ In the human umbilical vein endothelial cell, SIRT4 could inhibit inflammatory response through phosphatidylinositol 3 kinase/protein kinase B/nuclear factor kappa B (PI3K/Akt/NF‐κB) signaling pathway.^14^ Overexpression of SIRT4 inhibited the development of diabetes nephropathy through suppressing podocyte apoptosis.^15^, ^16^ Under the pathological state of insomnia, key brain regions such as hippocampus and locus coeruleus have higher levels of inflammation.^17^ Individuals with impaired expression of SIRT4 have impaired anti‐inflammatory ability and higher levels of apoptosis.^18^ Studies have shown that the locus coeruleus neurons in the brain have mitochondrial pressure during insomnia, leading to a decrease in the activity of SIRT3, an increase in the level of apoptosis, and a decrease in the number of neurons in the locus coeruleus.^19^ There is reason to speculate that neuronal apoptosis related to neuroinflammation is one of the important neural mechanisms of insomnia. As a functionally similar factor in the same family, SIRT4 has a high probability of also being located in key nuclei, playing a regulatory role in inhibiting neuroinflammatory responses and corresponding neuronal apoptosis, as well as in combating insomnia. However, if WY could improve neurological recovery in the insomnia rats through targeting SIRT4 has not been reported.

In this study, chlorophenylalanine (PCPA) induced insomnia rats were used in this study to investigate the influence of WY on neurological recovery of insomnia rats. We demonstrated that WY might improve the neurological recovery of insomnia rats through SIRT4. This study might provide a new insight for the treatment of insomnia through targeting SIRT4.

## 2 MATERIALS AND METHODS

### 2.1 Study medications

WY comprised of six medicinal herbs including *cooked aconite*, *dried ginger*, *roasted licorice*, *cinnamon twig*, *keel*, and *oyster*. The WY used in this study was obtained from the affiliated hospital of Jiangxi University of Traditional Chinese Medicine. The preparation method of WY was described below. Briefly, 10 g dried prescription of six herbs were decocted. The raw materials were mixed and crushed into small pieces. Six hundred milliliter water was added to raw materials and boiled for 2 h. The aqueous extract was filtered and stored at 4°C before use.

### 2.2 Cell culture and H_2_O_2_ treatment

Human neural stem cell line was used in this research for in vitro study. Roswell Park Memorial Institute (RPMI)‐1640 medium containing 5% fetal bovine serum (FBS) and 1% penicillin were used to cultivate cells in the incubator with 37°C and 5% CO_2_. The cells were cultured without FBS 24 h before use. After treatment with H_2_O_2_ (0.5%), WY (50 mg/mL), or siRNA‐SIRT4 (siSIRT4, 20 µM) for 4 h, cells were used for measurement of proliferation and apoptosis. siSIRT4 was designed and constructed by GenePharma Co., Ltd.

### 2.3 Establishment of insomnia animal model with PCPA induction

Sprague–Dawley male rats were used in this study, and these animals were obtained from the experimental animal center of Jiangxi University of Traditional Chinese Medicine. Intraperitoneal injection of PCPA (350 mg/kg) for two consecutive days was performed to construct insomnia rats model. Animals were divided into four groups (Control, PCPA, PCPA + WY, PCPA + WY + siSIRT4) with 10 rats in each group. The rats in the group control were treated with normal saline instead of PCPA. After establishment of insomnia rats with PCPA induction. The animals in the group PCPA + WY were treated with WY (10 g/kg) once a day through the gavage method. The animals in the group PCPA and control were treated with same amount of normal saline through the gavage method. The rats in the group PCPA + WY + siSIRT4 were treated with WY (10 g/kg) through gavage method and siSIRT4 (100 µM, 100 µL) with intravenous injection. After 6 days, the animals were killed and brain tissues were collected for TUNEL staining. All animal experiments have been approved by the Animal Ethical Committee of the affiliated hospital of Jiangxi University of Traditional Chinese Medicine (IACUC FJABR2021040701) and comply with Canadian Council on Animal Care guidelines. All experiments were performed in accordance with the guidelines for laboratory animal use.

### 2.4 Flow cytometry

Cells were cultured and treated as described in Section 2.2. Trypsin was used to digest cells. Cells were cultured with Annexin V‐fluorescein isothiocyanate (FITC) and propidium iodide for 20 min in the dark, and analyzed with a Cytomics_FC500 flow cytometer (Beckman).

### 2.5 Brdu staining

After fixation with using 4% paraformaldehyde (10 min), Triton X‐100 (1%) was used to incubate cells (15 min). Cells were blocked with 5% bovine serum albumin (BSA) for 20 min, and incubated with Brdu primary antibody (1:200, #5292; Cell Signaling) for 6 h. Then, cells were washed with phosphate‐buffered saline (PBS), and cultured with second antibody (1 h). Cell nucleuses were stained with 4',6‐diamidino‐2‐phenylindole (DAPI) (#KGA215‐50; Keygen), and a fluorescence microscope was used to capture images.

### 2.6 Morris water maze evaluation

A pool (35 cm high and 150 cm in diameter) filled with water was used in the Morris water maze. A platform (12 cm in diameter) was placed 1 cm below the water surface. The light of the experimental room is uniform, and the four walls have obviously different marks. This experiment adopted the scheme of 5‐day training (five trainings in each day for each animal) and 1‐day test. In each training, if the animal failed to find the platform within 1 min, it shall be guided to the platform manually and stayed for 20 s. After leaving the pool, each rat was immediately wiped dry with a towel and a heating lamp. At the 6th day, the rats were texted. Escape latency, swimming distance, times of crossing the original platform position were recorded.

### 2.7 Western blot analysis

Radio immunoprecipitation assay (RIPA) buffer containing the PMSF was used to dissolve brain tissues. After centrifugation, the supernatant was used for protein concentration detection. The same amount of protein was applied for sodium dodecyl sulfate–polyacrylamide gel electrophoresis (SDS‐PAGE). The proteins were transferred to a polyvinylidene fluoride (PVDF) membrane, and blocked with 5% skimmed milk (3 h). The membrane was washed with PBST, and incubated with related primary antibodies (1:800) overnight at 4°C. After washing with PBS twice, the membrane was incubated with second antibody (1:2000) for 3 h. Chemiluminescence with Thermo ECL Substrate (Bio‐Rad) was used to incubate the membrane, and ImageJ software was used to analyze the band gray. The antibodies used in this study were listed as follows: anti‐Bax antibody (ab32503; Abcam), anti‐nuclear factor erythroid 2‐related factor 2 (Nrf2) antibody (ab62352; Abcam), anti‐B‐Cell CLL/Lymphoma 2 (Bcl‐2) antibody (ab32124; Abcam), antitumor necrosis factor (TNF)‐α antibody (ab183218; Abcam), anti‐NLRP3 antibody (ab263899; Abcam), anti‐IL‐1β antibody (ab254360; Abcam), anti‐Glyceraldehyde 3‐phosphate dehydrogenase (GAPDH) antibody (ab9485; Abcam), and goat anti‐Rabbit IgG (ab205718; Abcam).

### 2.8 TUNEL staining

The slides were incubated with protease K without DNase first for 20 min. Hydrogen peroxide solution (1% H_2_O_2_) was used to incubate sections for 30 min. Terminal deoxynucleotidyl transferase (TdT) enzyme, Biotin‐dUTP, and biotin labeling solution (#C1091; Beyotime) were used to make TUNEL staining solution, which was used to stain sections for 2 h. Finally, streptavidin horseradish peroxidase (HRP) working solution and diaminobenzidine (DAB) chromogenic solution were used to incubate sections, and images were observed with a microscope.

### 2.9 Open‐field test evaluation

The rats were placed in an open field (100 × 100 × 50 cm), allowing rats to explore freely for 5 min. The following items such as autonomous activity time, horizontal activity distance, rearing, line crossing times, and time in the center zone were recorded.

### 2.10 Measurement of neurotransmitters

The brain tissues were collected after sacrifice. The levels of dopamine (#JL12965; Jianglai Bio), norepinephrine (#JL13428; Jianglai Bio), γ‐aminobutyric acid (GABA, #JL12343; Jianglai Bio), and 5‐hydroxytryptamine (5‐HT, #JL13043; Jianglai Bio) were detected with enzyme‐linked immunosorbent assay method using commercial kits.

### 2.11 Gene difference expression analysis

NanoDrop ND‐1000 was used to quantify RNA content, and RNA of each sample was transcribed into fluorescent complementary RNA (cRNA). The labeled cRNAs were hybridized onto the Whole Mouse Genome Oligo Microarray. Agilent Scanner G2505C was used to scan arrays. Acquired array images were analyzed with Agilent Feature Extraction software. Differentially expressed genes were identified through Fold Change filtering.

### 2.12 Statistical analysis

The data were shown as mean ± standard and analyzed with statistical package for the social sciences (SPSS) software. One‐way analysis of variance or Student's *t* test or were performed to analyze data. *p* < .05 was considered to be statistically different.

## 3 RESULTS

### 3.1 WY promoted neurological recovery in the insomnia rats through the Morris water maze evaluation

**Figure 1:**
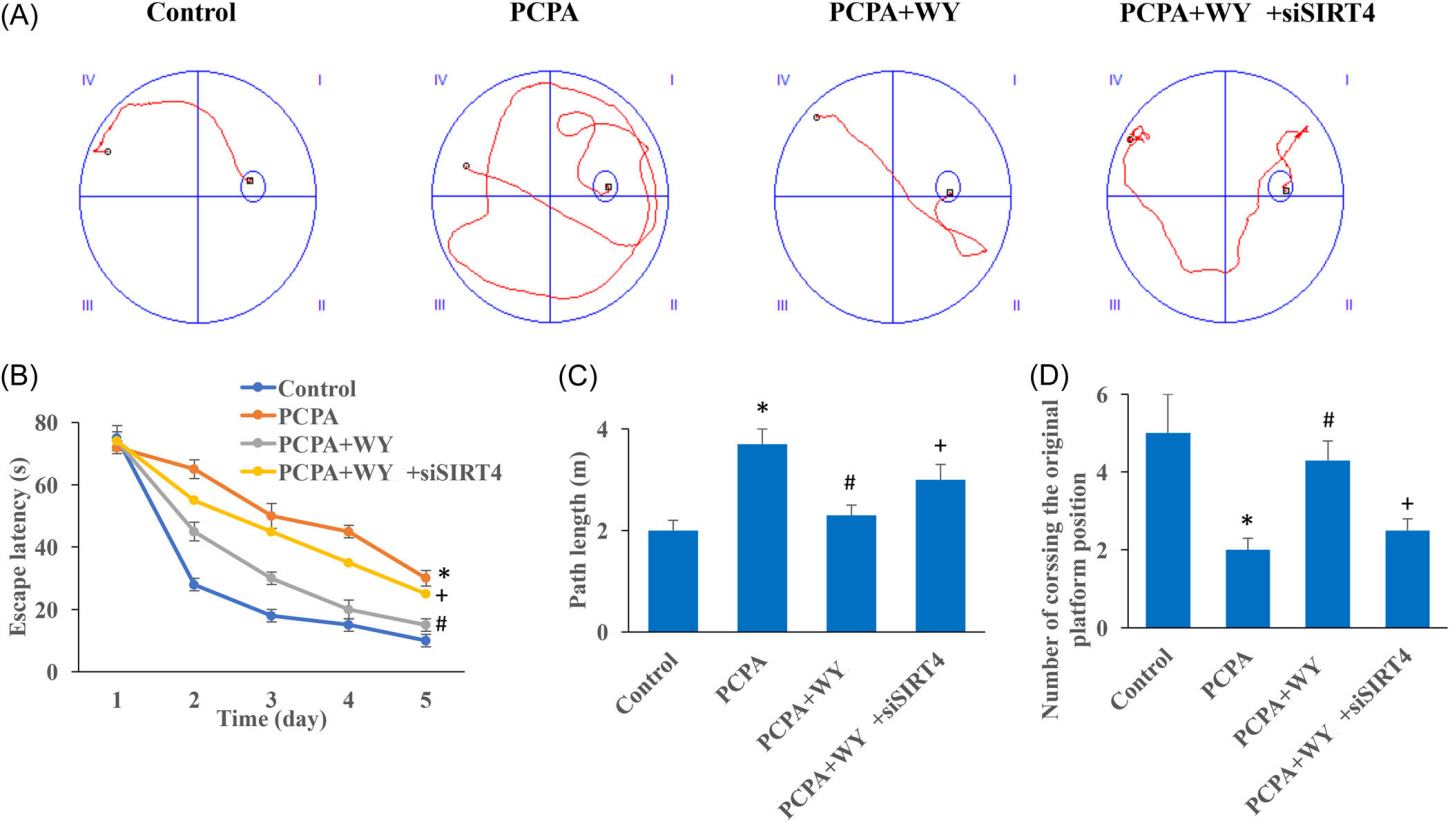
WY promoted neurological recovery in the insomnia rats through Morris water maze evaluation. (A) Morris water maze was applied to investigate the influence of WY on the neurological recovery of insomnia rats; (B) escape latency was analyzed in different groups; (C) path length was analyzed in different groups; (D) times of crossing the original platform position was analyzed in different groups. *n* = 5 and one‐way ANOVA followed Dunn's multiple comparisons test was performed for data analysis. ANOVA, analysis of variance; PCPA, chlorophenylalanine; WY, Warming Yang Strategy. *p < .05 compared to group control, ^#^ *p* < .05 compared to group PCPA, ^+^ *p* < .05 compared to group PCPA + WY.

Morris water maze was applied to investigate the influence of WY and SIRT4 on the neurological recovery of insomnia rats. We found that the escape latency (Figure 1A,B) and the swimming distance (Figure 1C) in the group PCPA were remarkably increased. However, after WY treatment, significant shorter escape latency and path length were observed compared to group PCPA (Figure 1A–C). In addition, knockdown of SIRT4 markedly reversed the influence of WY, and increased escape latency and swimming distance. After WY treatment, animals presented more times of crossing the original platform position compared to group PCPA (Figure 1D). However, siSIRT4 weakened the influence of WY on this item.

### 3.2 WY promoted neurological recovery in the insomnia rats through open‐field test evaluation

**Figure 2:**
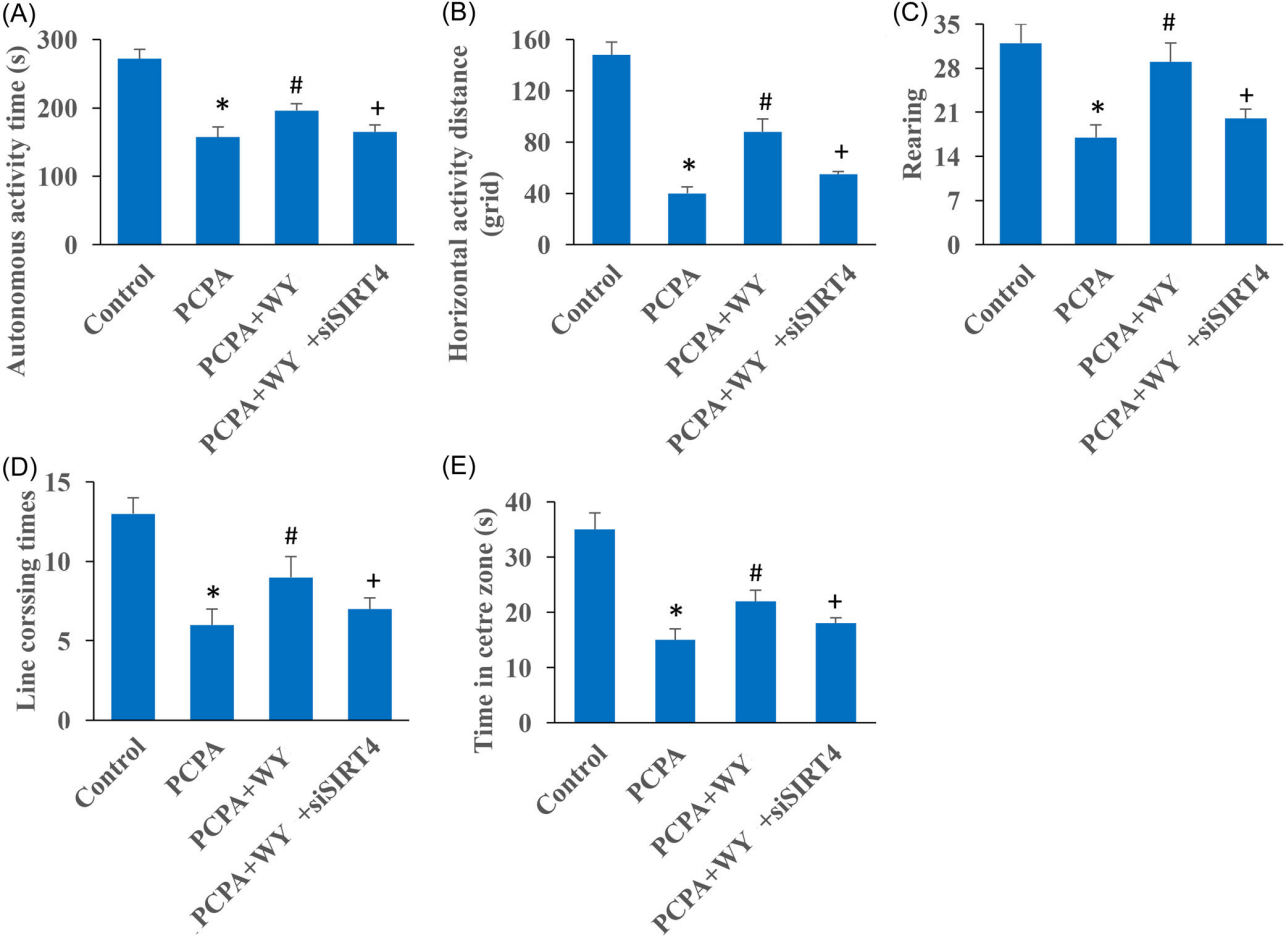
WY promoted neurological recovery in the insomnia rats through open field test evaluation. (A) Autonomous activity time was analyzed in different groups; (B) horizontal activity distance was analyzed in different groups; (C) rearing was analyzed in different groups; (D) line crossing times was analyzed in different groups; (E) time in the center zone was analyzed in different groups. *n* = 3 and one‐way ANOVA followed Dunn's multiple comparisons test was performed for data analysis. ANOVA, analysis of variance; PCPA, chlorophenylalanine; WY, Warming Yang Strategy. *p < .05 compared to group control, ^#^ *p* < .05 compared to group PCPA, ^+^ *p* < .05 compared to group PCPA + WY.

Open‐field test was also used to study the influence of WY and SIRT4 on the neurological recovery of insomnia rats. Several items including autonomous activity time (Figure 2A), horizontal activity distance (Figure 2B), rearing (Figure 2C), line crossing times (Figure 2D), and time in the center zone (Figure 2E), were investigated. After PCPA induction, the levels of these items were inhibited significantly (Figure 2A–E). However, WY administration could relieve the effects of PCPA induction. In addition, simultaneous treatment with siSIRT4 reversed the influence of WY, and suppressed the levels of autonomous activity time, horizontal activity distance, rearing, line crossing times, and time in the center zone (Figure 2A–E).

### 3.3 The increase of GABA, dopamine, 5‐HT, and norepinephrine caused by WY was suppressed by siSIRT4

**Figure 3:**
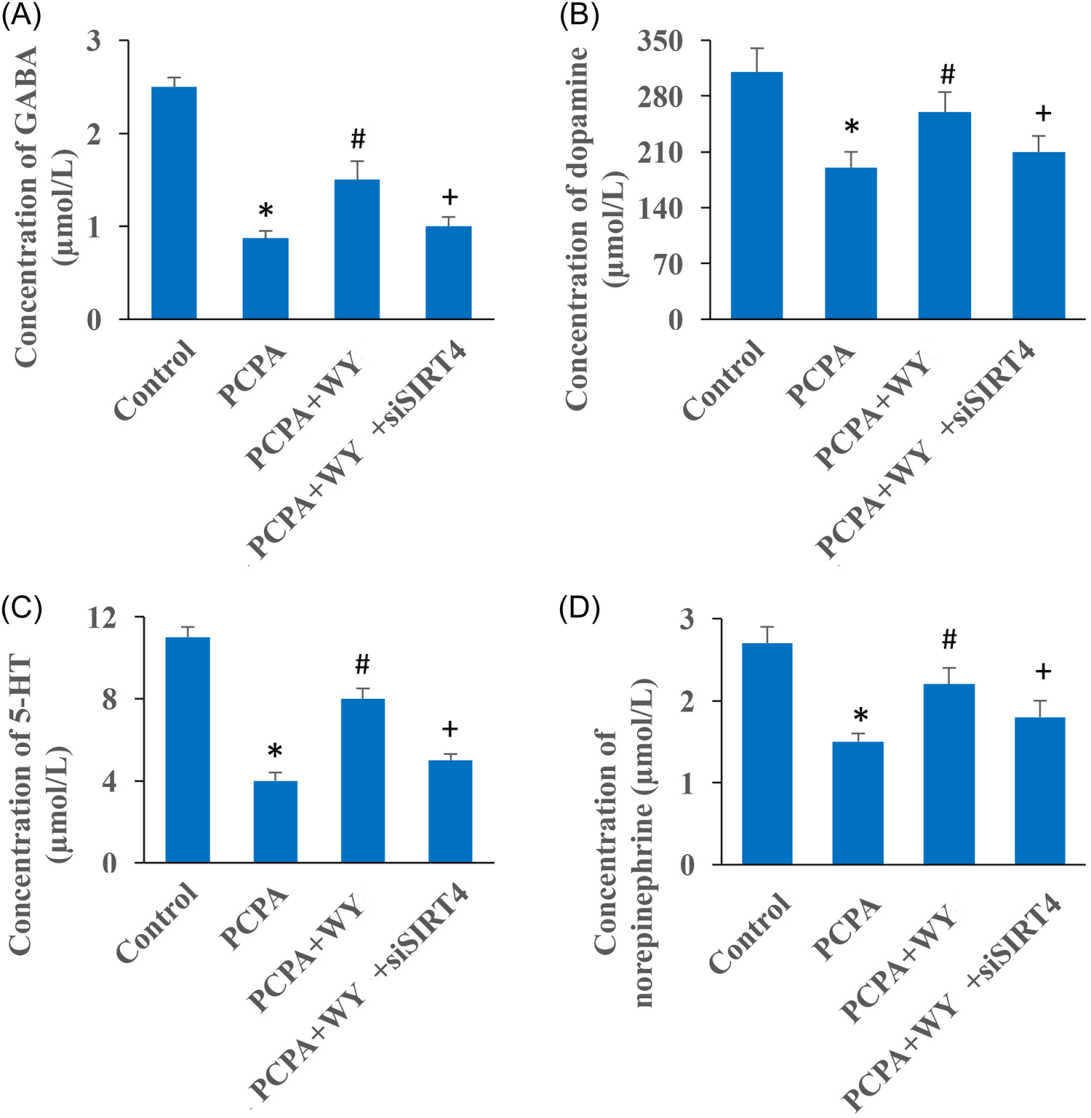
The increase of GABA, dopamine, 5‐HT, and norepinephrine caused by WY was suppressed by siSIRT4. (A) The concentration of GABA in different groups was measured. (B) The concentration of dopamine in different groups was measured. (C) The concentration of 5‐HT in different groups was measured. (D) The concentration of norepinephrine in different groups was measured. *n* = 3 and one‐way ANOVA followed Dunn's multiple comparisons test was performed for data analysis. 5‐HT, 5‐hydroxytryptamine; ANOVA, analysis of variance; GABA, γ‐aminobutyric acid; PCPA, chlorophenylalanine; WY, Warming Yang Strategy. *p < .05 compared to group control, ^#^ *p* < .05 compared to group PCPA, ^+^ *p* < .05 compared to group PCPA + WY.

The levels of GABA (Figure 3A), dopamine (Figure 3B), 5‐HT (Figure 3C), and norepinephrine (Figure 3D) in the brain tissues were remarkably suppressed after PCPA induction. However, the levels of these neurotransmitters were promoted after WY administration. In addition, the level promotion of GABA, dopamine, 5‐HT, and norepinephrine caused by WY were reversed after the knockdown of SIRT4.

### 3.4 The decrease of apoptosis and inflammation factors expression induced by WY was promoted by siSIRT4

**Figure 4:**
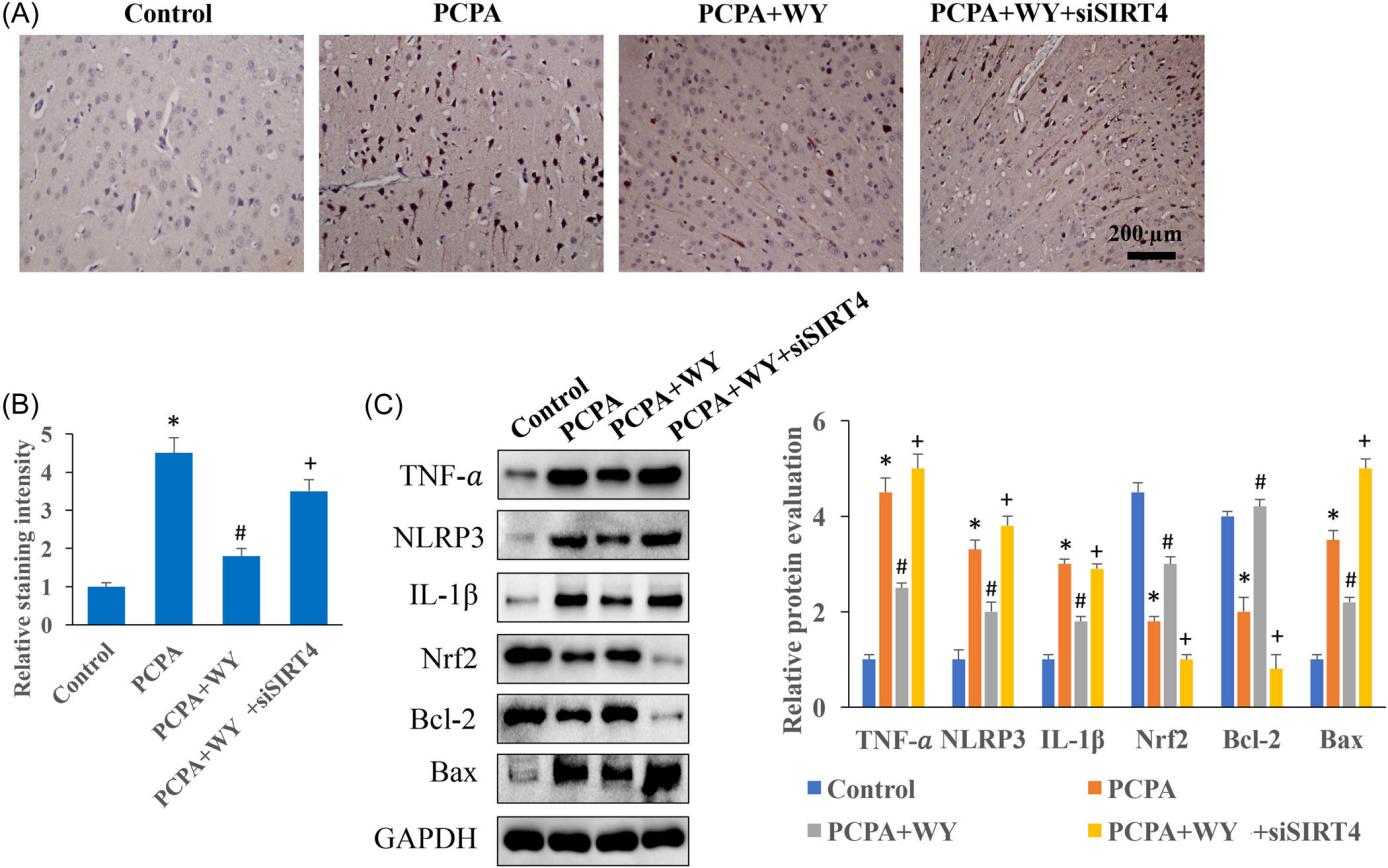
The decrease of apoptosis and inflammation factors expression induced by WY was promoted by siSIRT4. (A) The apoptosis in the tissues was evaluated with TUNEL staining (Magnification: ×200). (B) TUNEL staining intensity was analyzed. (C) The influence of WY and siSIRT4 on apoptosis and inflammation was evaluated with western blot analysis. *n* = 3 and one‐way ANOVA followed Dunn's multiple comparisons test was performed for data analysis. ANOVA, analysis of variance; IL, interleukin; PCPA, chlorophenylalanine; TNF, tumor necrosis factor; WY, Warming Yang Strategy. *p < .05 compared to group control, ^#^ *p* < .05 compared to group PCPA, ^+^ *p* < .05 compared to group PCPA + WY.

**Figure 5:**
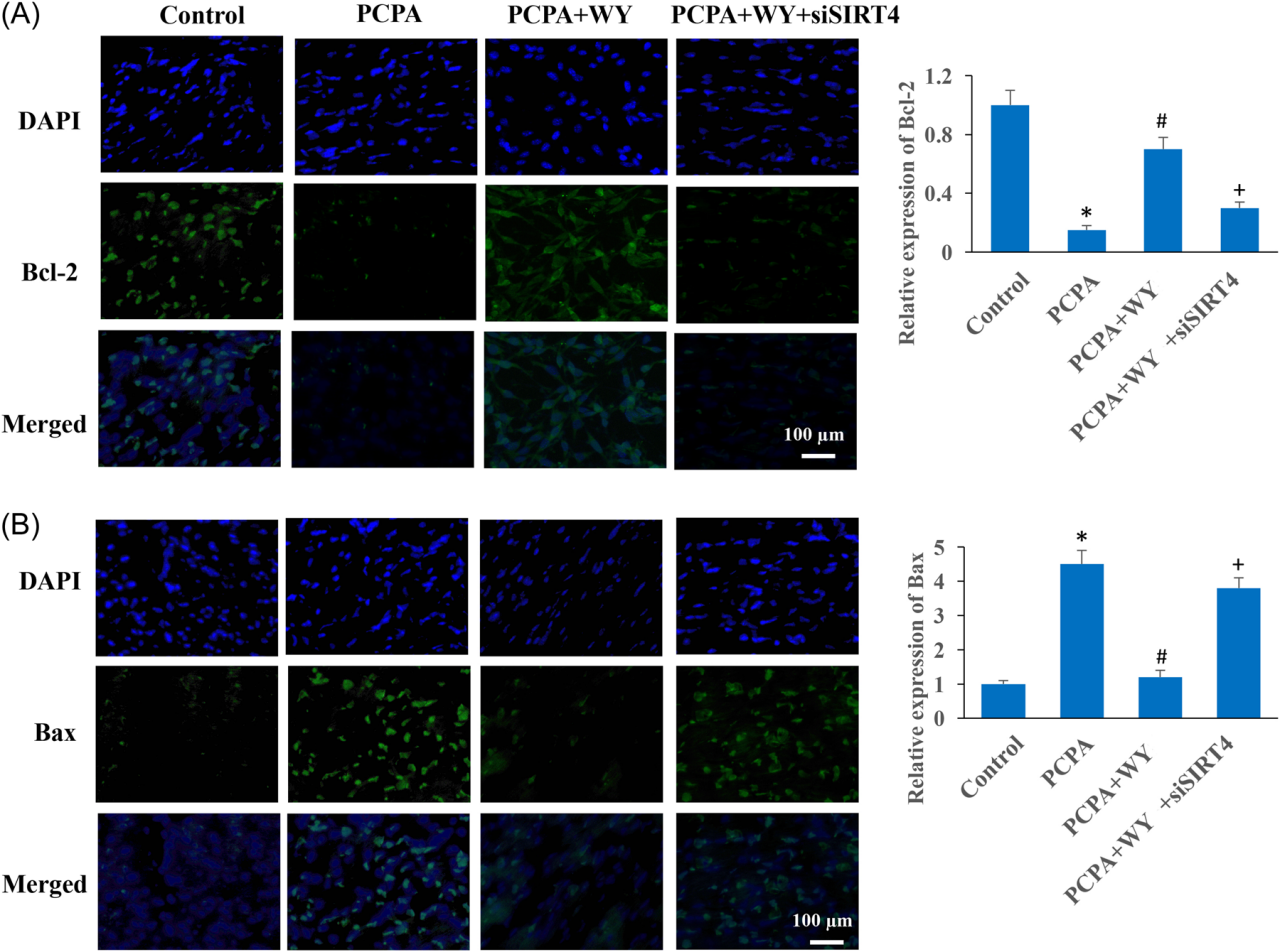
The influence of WY on Bcl‐2 and Bax was reversed by siSIRT4. (A) The expression level of Bcl‐2 in vivo was validated with immunohistochemistry staining (Magnification: ×400). (B) The expression level of Bax in vivo was validated with immunohistochemistry staining (Magnification: ×400). *n* = 3 and one‐way ANOVA followed Dunn's multiple comparisons test was performed for data analysis. ANOVA, analysis of variance; PCPA, chlorophenylalanine; WY, Warming Yang Strategy. *p < .05 compared to group control, ^#^ *p* < .05 compared to group PCPA, ^+^ *p* < .05 compared to group PCPA + WY.

The apoptosis intensity was evaluated with TUNEL staining and measurement of apoptosis‐related proteins. We found that the significant increase of apoptosis intensity in the group PCPA was decreased in the group PCPA + WY (Figure 4A,B). However, the decreased apoptosis was reversed by knocking down siSIRT4. In addition, WY could inhibit inflammation response by decreasing TNF‐α, NLRP3, and IL‐1β, but increasing Nrf2 compared to group PCPA. Meanwhile, inhibition of apoptosis by WY was observed through promoting Bcl‐2, and inhibiting Bax compared to group PCPA (Figure 4C). The regulatory effects of WY on apoptosis and inflammation response were markedly reversed by siSIRT4. The expression levels of Bcl‐2 and Bax in vivo were also validated with immunohistochemistry staining (Figure 5A,B), similar findings to western blot analysis were observed.

### 3.5 The promotion of cell viability caused by WY was suppressed by siSIRT4 in vitro

**Figure 6:**
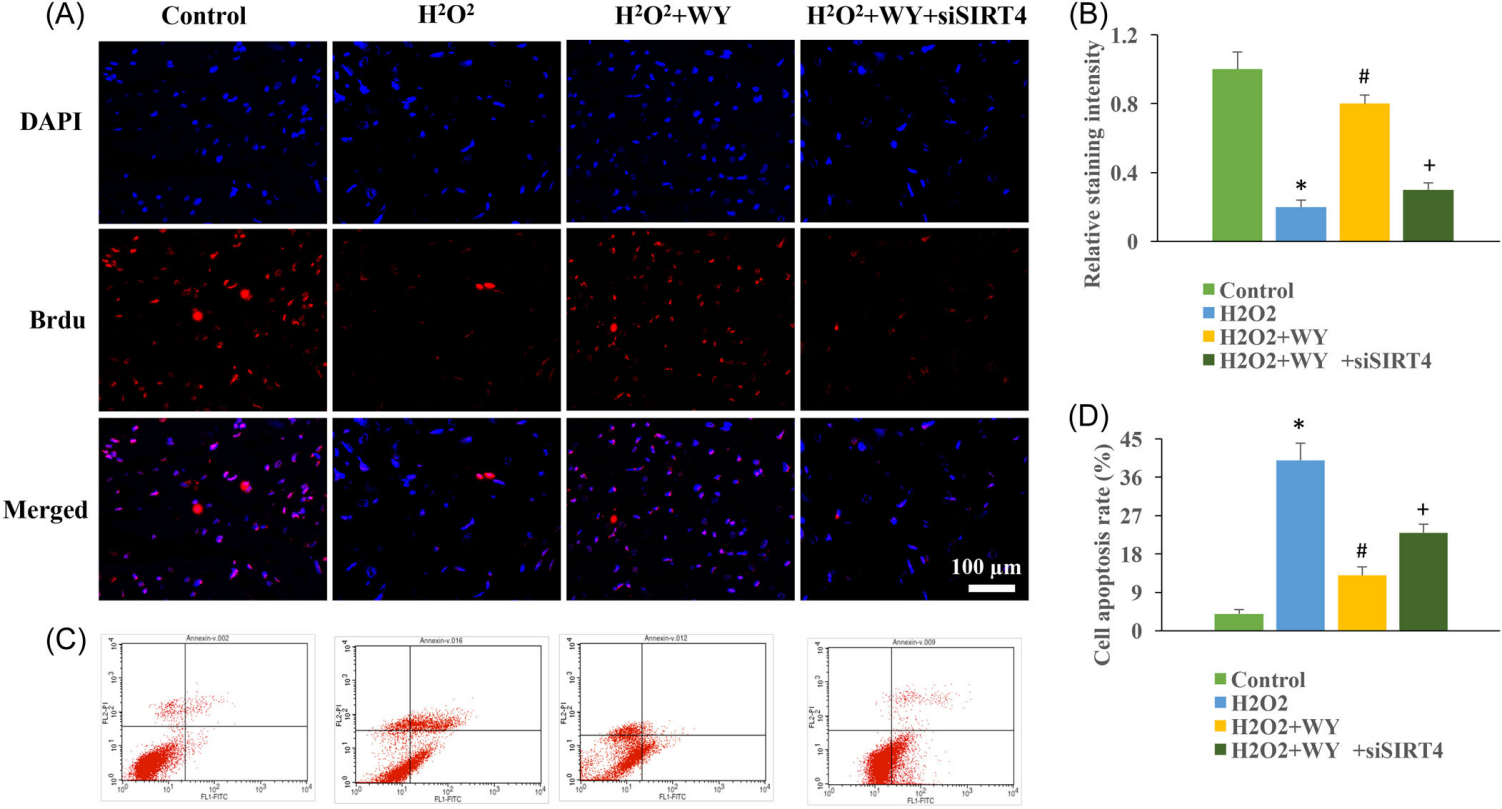
The promotion of cell viability caused by WY was suppressed by siSIRT4 in vitro. (A) Brdu staining was used to evaluate cell proliferation (Magnification: ×400). (B) The cell proliferation was analyzed. (C) The cell apoptosis was measured with flow cytometry. (D) The cell apoptosis was analyzed. *n* = 3 and one‐way ANOVA followed Dunn's multiple comparisons test was performed for data analysis. ANOVA, analysis of variance; WY, Warming Yang Strategy. *p < .05 compared to group control, ^#^ *p* < .05 compared to group H_2_O_2_, ^+^ *p* < .05 compared to group H_2_O_2_ + WY.

The influence of WY and SIRT4 on neurocyte viability was also evaluated with Brdu staining and flow cytometry. We found that after treatment H_2_O_2_, cell proliferation (Figure 6A,B) was inhibited, and cell apoptosis (Figure 6C,D) was promoted. The cell viability was promoted after WY compared to group control (Figure 6A–D). However, after transfection with siSIRT4, the influence of WY on cell proliferation and apoptosis was reversed (Figure 6A–D).

### 3.6 Bioinformatics analysis

**Figure 7:**
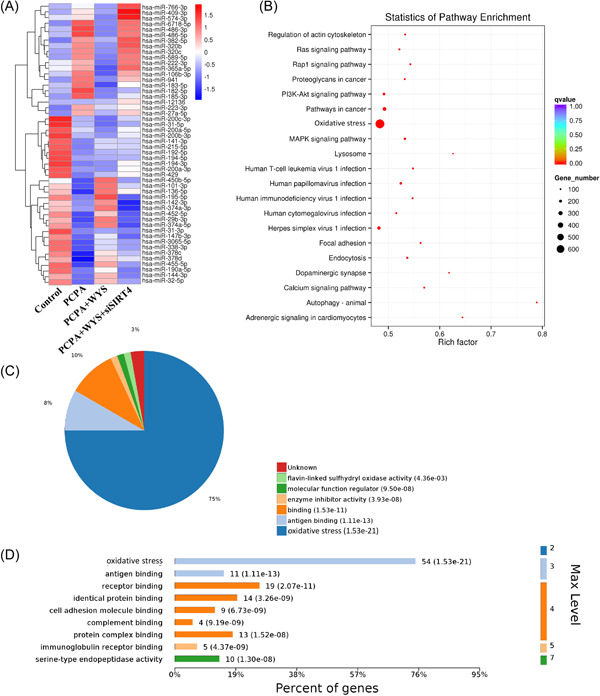
Bioinformatics analysis among different groups. (A) MicroRNA array analysis of microRNA expression. (B) Pathway enrichment analysis was performed to investigate the influence of WY on PCPA. (C) Differential expression genes between group PCPA and PCPA + WY were analyzed. *n* = 3. ANOVA, analysis of variance; PCPA, chlorophenylalanine.

MicroRNA array was performed to observe the expression differences of microRNAs among different groups (Figure 7A). The minimum expression difference of microRNAs was observed between group control and PCPA + WY. Remarkable microRNAs expression differences were found in group PCPA and PCPA + WY + siSIRT4 compared with group control or PCPA + WY (Figure 7A). In addition, pathway enrichment analysis was performed to investigate the influence of WY on PCPA (Figure 7B). Differential expression genes between group PCPA and PCPA + WY were analyzed. Genes related with oxidative stress might be influenced by WY (Figure 7C,D).

## 4 DISCUSSION

Research have shown that good sleep can help restore physical function, consolidate memory, prevent Alzheimer's disease, and enhance the immune function of the body.^20^ Insomnia may lead to neurological impairment symptoms such as memory loss.^21^ Insomnia patients are usually accompanied by varying degrees of depression, anxiety, and other psychotic symptoms. Insomnia has become a social problem threatening people's health.^22^

PCPA is an inhibitor of 5‐HT synthesis, and PCPA can inhibit the synthesis of 5‐HT in rat brain, resulting in the disappearance of sleep circadian rhythm and even almost complete insomnia.^23^, ^24^ In this study, the levels of neurotransmitters including GABA, dopamine, 5‐HT, and norepinephrine were inhibited significantly after PCPA induction (Figure 3). Meanwhile, neurological function was damaged in the group PCPA evaluating with Morris water maze and open field test (Figures 1, 2). GABA is a very important inhibitory neurotransmitter in the brain physiological activities. GABA, dopamine, 5‐HT, and norepinephrine have been proved to be closely related with the maintenance of electroencephalogram wakefulness and behavioral arousal. We demonstrated that the increase of neurotransmitters by WY were decreased by knocking down SIRT4 (Figure 3).

Abnormally elevated inflammatory response and accompanying neuronal apoptosis may constitute to the central mechanism of insomnia.^5^ There is a certain correlation between insomnia and inflammatory level. Many depression and anxiety studies have found that sleep duration, insomnia, and inflammatory markers levels are correlated.^25^ It was reported that insomnia could lead to the increase of inflammatory factors. Meanwhile the promotion of Bax, downregulation of Bcl‐2, and increase of apoptosis rate were observed after insomnia.^10^

Previous studies indicated that silencing of SIRT4 exacerbated the expression of pro‐inflammatory cytokines such as IL‐1β, IL‐6, IL‐8, cyclooxygenase‐2 (COX2), matrix metalloproteinase‐9 (MMP‐9), and intercellular adhesion molecule‐1 (ICAM‐1).^15^ The upregulation of these genes is related to inflammation and vascular remodeling. Meanwhile, overexpression of SIRT4 prevented NF‐κB nuclear translocation, and inhibited inflammation through interfering with NF‐κB.^15^, ^26^ The modulating roles of SIRT4 in nervous system diseases have also been reported. The recovery of olfactory function was associated with the increase of SIRT4 expression.^27^ SIRT4 can inhibit the anti‐inflammatory activity of Tregs infiltrating the spinal cord after injury.^28^ In the present study, we proved that the decreased TNF‐α, NLRP3, and IL‐1β caused by WY were elevated by siSIRT4. These results indicated that the regulation of inflammation by WY might be achieved through SIRT4.

There were some limitations in our study. For example, the specific functioning fraction in the WY remains unclear. Second, the further signaling pathway influenced by SIRT4 needs to be validated. Moreover, whether SIRT4 could be a predicting marker of neurological disease requires further analysis.

## 5 CONCLUSION

In summary, we found that WY might promote neurological recovery in the insomnia rats through increasing neurotransmitters, inhibiting apoptosis and inflammation response. This study might provide a new therapeutic strategy for the treatment of insomnia through targeting SIRT4.

## AUTHOR CONTRIBUTIONS

**Yuanyuan Liu**: Project administration; resources; validation. **Kaihua Rao**: Methodology; project administration. **Zhengfeng Li**: Project administration; software; supervision; Chunhua Huang: Project administration; funding support; manuscript writing.

## CONFLICT OF INTEREST STATEMENT

The authors declare no conflict of interest.

## ETHICS STATEMENT

All animal protocols have been approved by the Animal Ethical Committee of the affiliated hospital of Jiangxi University of Chinese Medicine (Approval number: IACUC FJABR2021040701), and all experiments were performed in accordance with the guidelines for laboratory animal use.

## Data Availability

Data generated or analyzed during this study are available from the corresponding author upon reasonable request.
